# Numerical Analysis of Fatigue Crack Growth Path and Life Predictions for Linear Elastic Material

**DOI:** 10.3390/ma13153380

**Published:** 2020-07-30

**Authors:** Abdulnaser M. Alshoaibi, Yahya Ali Fageehi

**Affiliations:** Department of Mechanical Engineering, Jazan University, P. O. Box 706, Jazan 45142, Saudi Arabia; yfageehi@jazanu.edu.sa

**Keywords:** fatigue crack growth, mixed mode, MCTS, ANSYS, LEFM, fatigue life

## Abstract

The main objective of this work was to present a numerical modelling of crack growth path in linear elastic materials under mixed-mode loadings, as well as to study the effect of presence of a hole on fatigue crack propagation and fatigue life in a modified compact tension specimen under constant amplitude loading condition. The ANSYS Mechanical APDL 19.2 is implemented for accurate prediction of the crack propagation paths and the associated fatigue life under constant amplitude loading conditions using a new feature in ANSYS which is the smart crack growth technique. The Paris law model has been employed for the evaluation of the mixed-mode fatigue life for the modified compact tension specimen (MCTS) with different configuration of MCTS under the linear elastic fracture mechanics (LEFM) assumption. The approach involves accurate evaluation of stress intensity factors (SIFs), path of crack growth and a fatigue life evaluation through an incremental crack extension analysis. Fatigue crack growth results indicate that the fatigue crack has always been attracted to the hole, so either it can only curve its path and propagate towards the hole, or it can only float from the hole and grow further once the hole has been lost. In terms of trajectories of crack propagation under mixed-mode load conditions, the results of this study are validated with several crack propagation experiments published in literature showing the similar observations. Accurate results of the predicted fatigue life were achieved compared to the two-dimensional data performed by other researchers.

## 1. Introduction

The assessment of FCG and the fracture toughness of materials is commonly performed using the CT specimen (ASTM 2013). In fatigue crack growth (FCG) studies, the specimen geometry typically investigates the ratio of minimum stress to maximum stress (*R*) [1,2]. The compact tension CT specimen has the beneficial effects of a relatively smaller material volume and comparatively less stress for FCG evaluation [3]. Practical structures are almost subjected to many loading types like tension, shear and torsion resulting in a mixed-mode interaction. Accordingly, the stress state ahead of a crack is commonly based on mixed-mode I/II type of interactions, indicating the magnitude of the stresses at the crack tip. Therefore, cracks can grow in the skin of aircraft fuselages and may subject to mixed-mode type of loading. Generally, crack initiation and propagation must be associated with the governing stress intensity factors (SIFs) in a complicated state [4,5,6,7]. Fatigue crack analysis is essential in different fields of engineering since fatigue cracks are one among the main sources of catastrophic fracture failures. It is important to guarantee the durability of crucial structures and establishing safety of structures in working conditions. Accordingly, in many industries, the precise crack path prediction and estimation of fatigue life are of primary importance in terms of the reliability requirement. Experimental studies are required for fatigue analysis in various applications, such as aerospace manufacturing and aviation industry, but due to high costs, precise computational methods are required for crack propagation analysis to predict the path of crack growth and fatigue lifetime in both static and dynamic loading conditions [8]. The scientific literature on FCG activity concentrate on mode-I crack, while cracks and defects in real-world structures are typically mixed-mode (I–II) cracking and the cracks in actual engineering components (e.g., aircraft, pressure vessels component, high-pressure pipelines, etc.) seem to be some combination of mixed-mode (mode I, mode II and mode III) [9]. Practically, mixed mode can also be encountered, e.g., in the case of shafts attached to turbines subjected to abrupt change in loading directions. Generally, the initiation process in crack formation is due to the plastic strain resulting from cyclic tension and the propagation of cracks happen due to the presence of tensile stress in matrix. In spite of the presence of local stresses, the compressive loads cannot cause the fatigue crack initiation [10]. In order to prevent failures of fatigue, extensive work was done to develop efficient models to evaluate the FCG and fatigue life. There are several experimental models proposed, but it is usually time consuming and expensive to conduct the experimental procedures. The simulation technique involving the numerical analysis and usage of ANSYS APDL.19.2 extended finite-element approach is an appropriate way of minimizing experimental work, time and cost. Stress intensity factors (SIFs) are used to describe the displacement and stresses of the crack front and consequently the evaluation of FCG under the assumption of linear elastic fracture mechanics (LEFM). As soon as the crack is growing, the SIF will shift to a critical range in which the structure deformation initiates leading to the failure process. The majority of fatigue-crack problems reported in the literature till date are using various methods of analysis in terms of two- and three-dimensional simulation for simple as well as complex geometries [8,11,12,13,14,15,16,17,18], etc.. Because of the complexity of applied loads and the geometry specification, mixed mode (I/II) are the usual types of loads that depend on those used in fatigue life predictions [19,20,21,22]. Hence, this work utilizes the XEFM employed by ANSYS APDL 19.2 to determine the influence of the hole position in the crack growth direction, stress intensity factors and also fatigue life of the modified compact tension specimen (MCTS) specimen. The main motivation of this work was to bring some contribution on the use of the ANSYS as an alternative method for the simulation of fatigue crack propagation problems under mixed-mode loading and to provide monitoring of the crack growth trajectory in the cases of the presence of holes in the geometry.

## 2. Numerical Predication of Mixed-Mode Fatigue Life

The latest innovation by Ansys Mechanical APDL 19.2 is the “smart crack growth” feature which was applied in the present study. By using this feature in Ansys Mechanical APDL (version 19.2, Ansys, Inc., Canonsburg, PA, USA), engineers have recently employed the modern unstructured mesh method (UMM) to minimize pre-processing times by using the automatically generated all-tetrahedral mesh for the crack fronts and achieve the same high-fidelity results as a simulation with the ideal hex mesh configuration. Meshing time has been reduced from a few days to a few minutes. With several clicks, a SMART simulation can be set up, eliminating long pre-processing sessions. UMM is more flexible and simpler to use than any previous technology for fracture simulation. Automatic remeshing is automatically done in the vicinity of the crack tip as well as refines calculations in the most needed regions with higher stresses for better visualization and accurate results calculation without requiring the engineer’s intervention. Another feature for the “Smart Crack Growth” is the introducing of the “premeshed crack.” The mesh around the crack tip should be refined using the sphere of influence method around the geometric edge going through thickness. Within the premesh crack object, the node sets created previously are allocated to the crack front and the crack top and bottom faces. Reference is made to the crack coordination system and the number of contours for the solution is set to 5. There are “loops” through the mesh around the crack point that are used by the integration of the crack tip region with the strain energy to determine the stress factor. The fracture mechanics method avoids the stress singularities at the crack tip in the analysis.

Three methods were used mainly to demonstrate material fatigue assessments, namely, the technique of fracture mechanics [23], the method of strain-life introduction independently [24] and the method involving stress-life [25].

**Figure 1:**
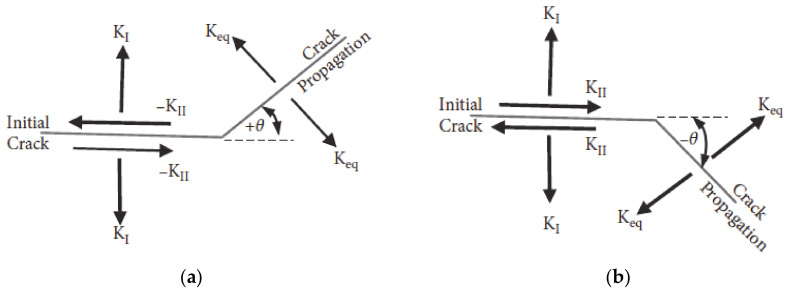
Prospects of the angle of crack propagation (**a**) *K_II_* › 0 and (**b**) *K_II_* ‹ 0.

In order to explain the cracking tip by individual SIFs, the first technique for predicting fatigue life was employed. Therefore, the fatigue crack growth direction must be precisely calculated for the evaluation of fatigue life. As a result, the maximum tangent stress theory was used to determine the angle of crack growth [15,26,27] as:

(1) $$\theta =2\mathrm{arc}\tan{\left(\frac{1}{4}\frac{K_{I}}{K_{\mathrm{II}}}+\frac{1}{4}\sqrt{{\left(\frac{K_{I}}{K_{\mathrm{II}}}\right)}^{2}+8}\right)}_{\mathrm{for}\;K_{\mathrm{II}\;}<\;0}$$

(2) $$\theta =2\mathrm{arc}\tan{\left(\frac{1}{4}\frac{K_{I}}{K_{\mathrm{II}}}-\frac{1}{4}\sqrt{{\left(\frac{K_{I}}{K_{\mathrm{II}}}\right)}^{2}+8}\right)}_{\mathrm{for}\;K_{\mathrm{II}\;}>\;0}$$

where *θ* is the crack growth angle and *K_I_* and *K_II_* are the first and second mode of stress intensity factor, respectively. The crack growth angles according to the sign of the second mode of stress intensity factor, *K_II_*, are displayed in Figure 1.

Prediction of FCG by utilizing the corresponding SIFs is the most commonly utilized technique for mixed-mode fatigue loading structures. Tanaka [20] derived an innovative law, so-called power law, for the modified Paris law equation for the determination of crack growth in response to fatigue with respect to parameter the equivalent stress intensity factor ($∆K_{eq}$). It is indicated as:

(3) $$\frac{da}{dN}=C\left(∆K_{eq}\right)$$

where *a* is the crack length, *N* is the number of cycles, *C* is the Paris constant (mm/cycle) and *m* is the Paris exponent.

The quantitative nature of fatigue life cycles can be calculated using Equation (3) for an increase in crack length as:

(4) $$\overset{∆a}{\underset{0}{\int }}\frac{da}{C{\left(∆K_{eq}\right)}^{m}}=\overset{∆N}{\underset{0}{\int }}dN=∆N$$

## 3. Numerical Results and Discussion

**Figure 2:**
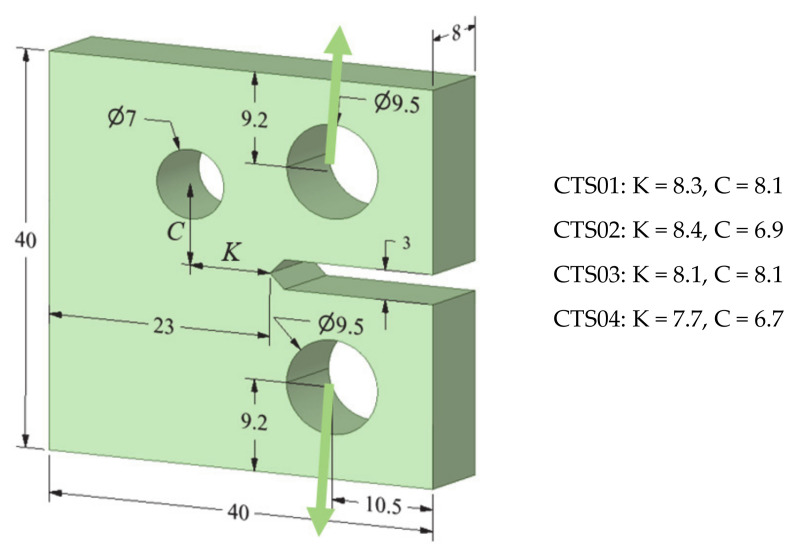
Geometrical description of the modified compact tension specimen (MCTS) (dimensions in mm).

The experimental work conducted by [28] on four SAE 1020 carbon steel hole MCTS specimens is used in this study of the MCTS to demonstrate an ability to predict the crack propagation trajectories and the fatigue life in mixed-mode under constant amplitude load conditions. The geometric dimensions of modified CT specimen are shown in Figure 2. The main hole diameter is 7 mm, positioned at horizontal and vertical distances *K* and *C,* respectively, from the crack initiation position as shown in Figure 2. The properties of the considered material are shown in Table 1. The simulation is performed under fatigue loading with the assumption that the tested material is isotropic and linear elastic with load ratio of *R* = 0.1.

**Figure 3:**
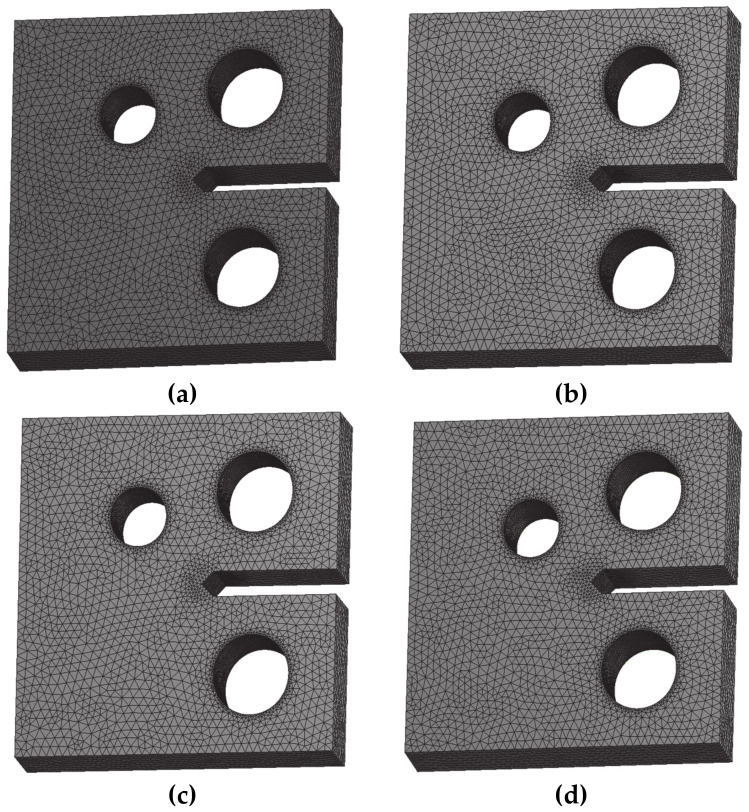
Finite element model for the MCTS, (**a**) CT01, (**b**) CT02, (**c**) CT03 and (**d**) CT04.

ANSYS Workbench software is used to generate the mesh for the four specimens with following mesh densities (Table 2). Figure 3 shows the initial FE model of the specimen along with its boundary condition.

**Figure 4:**
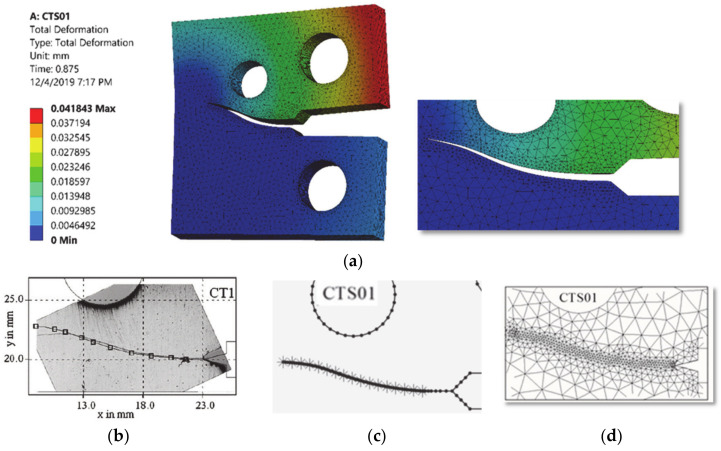
Predicted crack growth direction of CTS01: (**a**) current study result, (**b**) experimental and numerical results of [28], (**c**) BemCracker2D [16] and (**d**) Quebra2D [16].

**Figure 5:**
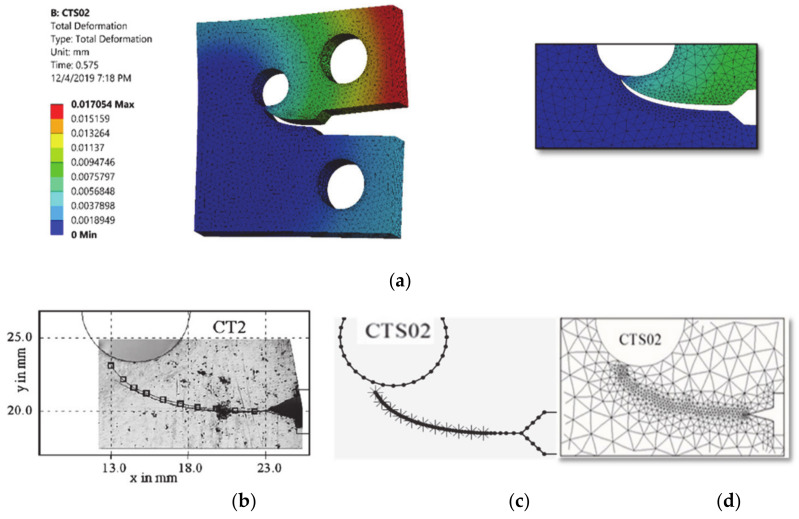
Predicted crack growth direction of CTS02: (**a**) current study result, (**b**) experimental and numerical results of [28], (**c**) BemCracker2D [16] and (**d**) Quebra2D [16].

**Figure 6:**
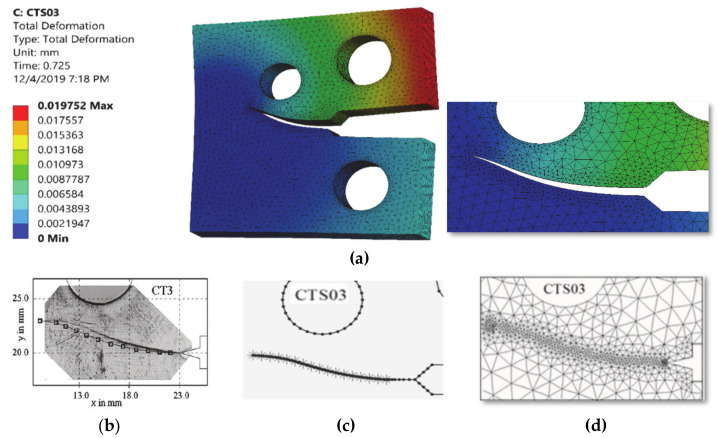
Predicted crack growth direction of CTS03: (**a**) current study result, (**b**) experimental and numerical results of [28], (**c**) BemCracker2D [16] and (**d**) Quebra2D [16].

**Figure 7:**
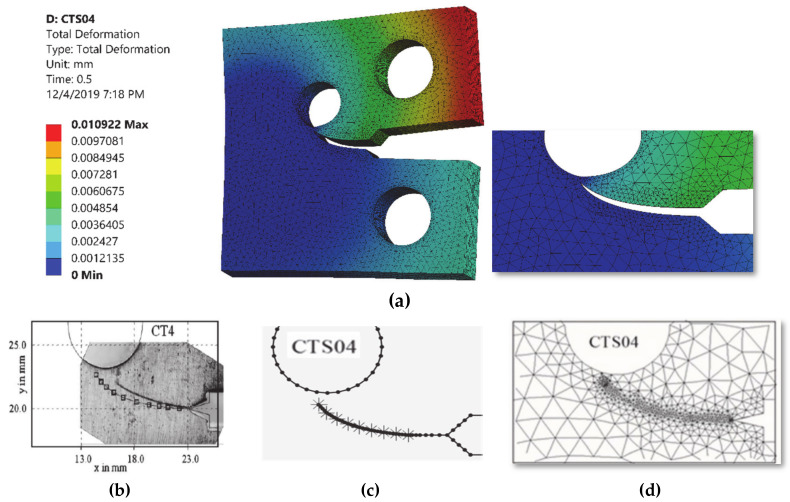
Predicted crack growth direction of CTS04: (**a**) current study result, (**b**) experimental and numerical results of [28], (**c**) BemCracker2D [16] and (**d**) Quebra2D [16].

**Figure 8:**
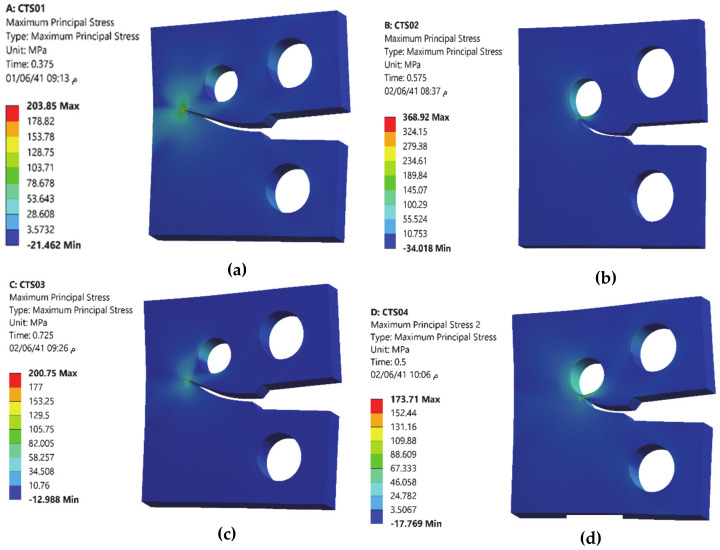
Maximum principal stress distribution for different configuration of CTS, (**a**) CT01, (**b**) CT02, (**c**) CT03 and (**d**) CT04.

**Figure 9:**
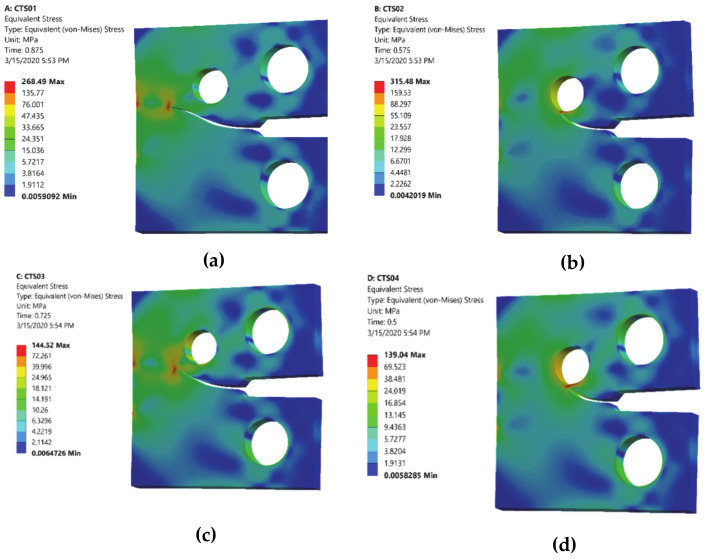
Equivalent Von Mises stress distribution for different configuration of CTS, (**a**) CT01, (**b**) CT02, (**c**) CT03 and (**d**) CT04.

The comparisons of simulated and experimental and numerical crack path performed by [28] and [16] are shown for CT01, CT02, CT03 and CT04 in Figure 4, Figure 5, Figure 6 and Figure 7, respectively. The modified CTS holes were explicitly designed to manipulate the crack direction. As shown in the figures, the crack growth paths are almost identical to the path predicted experimentally and numerically [28] and [16], using boundary element method (BEM) with BemCracker2D software (which is a special purpose educational program for simulating two-dimensional crack growth based on the dual boundary element method, written in C++ with a MATLAB graphic user interface developed by [16,28] and finite element method with Quebra2D (which is a finite element based software developed by [16,28]). Also, it is worth visualizing the maximum principle stress and the equivalent stress distribution of Von Mises of mentioned four different CTS configurations as shown in Figure 8 and Figure 9, respectively. The Von Mises yield criterion is used to compute yielding of materials under multiaxial loading conditions depending on the maximum and minimum principal stress and also the shear stress. As these two figures explicitly demonstrate, there is a significant association between the maximum principal stress and Von Mises stress in the four different models of the CTS.

The stress intensity factor (SIF) is the essential parameter for life assessment. Numerous handbooks can also provide analytical computation of the SIF for the standard CT geometry. The formulated analytical stress intensity factor solution for the standard CT geometry is expressed as follows [29,30,31]:

(5) $$K_{I}=\frac{Pf\left(a/w\right)}{t\sqrt{w}}.\;$$

where *P* is the applied load, *t* is the geometry thickness and *f(a/w)* is referred to as either the correction factor or the dimensionless SIF, which depends on the length of the crack (*a*) to the width of the specimen (*w*) ratio and is defined from [31,32] and [33] as:

(6) $$f\left(\frac{a}{w}\right)=\frac{2+\frac{a}{w}}{{\left(1-\frac{a}{w}\right)}^{\frac{3}{2}}}\left[0.886+4.64\left(\frac{a}{w}\right)-13.32{\left(\frac{a}{w}\right)}^{2}+14.72{\left(\frac{a}{w}\right)}^{3}-5.6{\left(\frac{a}{w}\right)}^{4}\right]$$

This correction factor manual approach is provided for loading mode I. The presence of the hole in this modified specimen has produced a curved crack trajectory. Due to the curved crack trajectory, the solution for correction factor given under Equation (5) is no longer valid. At this point, we can see the main advantages of mixed-mode crack growth with numerical analysis like using XFEM. ANSYS can achieve accurate predicted values for *f(a/w)* different from manual solutions obtained for regular CT specimens. At each step of the crack growth, mode I SIF’s (*K_I_*) is collected from ANSYS results and substituted in Equation (5) to obtain the dimensionless stress intensity factor *f(a/w)*. The polynomial of the fourth degree is fitted with the stress intensity factor in the following equations for CT01, CT02, CT03 and CT04, respectively.

(7) $$CT01\;K_{I}=\frac{P}{wt}\;\sqrt{\pi a}\;\left(209.07{\left(a/w\right)}^{4}-1572.1{\left(a/w\right)}^{3}+235.85{\left(a/w\right)}^{2}-58.901\left(a/w\right)+10.44\right)$$

(8) $$CT02\;K_{I}=\frac{P}{wt}\;\sqrt{\pi a}\;\left(-106.8{\left(a/w\right)}^{4}+175.9{\left(a/w\right)}^{3}-84.369{\left(a/w\right)}^{2}+24.324\left(a/w\right)+2.5509\right)$$

(9) $$CT03\;K_{I}=\frac{P}{wt}\;\sqrt{\pi a}\;\left(300.81{\left(a/w\right)}^{4}-511.98{\left(a/w\right)}^{3}+325.41{\left(a/w\right)}^{2}-80.886\left(a/w\right)+12.372\right)$$

(10) $$CT04\;K_{I}=\frac{P}{wt}\;\sqrt{\pi a}\;\left(1130.1{\left(a/w\right)}^{4}-1572.1{\left(a/w\right)}^{3}+829.56{\left(a/w\right)}^{2}-184.61\left(a/w\right)+20.36\;\right)$$

The obtained SIF data set can be used to construct an easy-to-use formula through the general linear regression technique, expressing the SIF as a function of the interested crack and contact parameters and facilitating the evaluation of the crack propagation behaviour.

**Figure 10:**
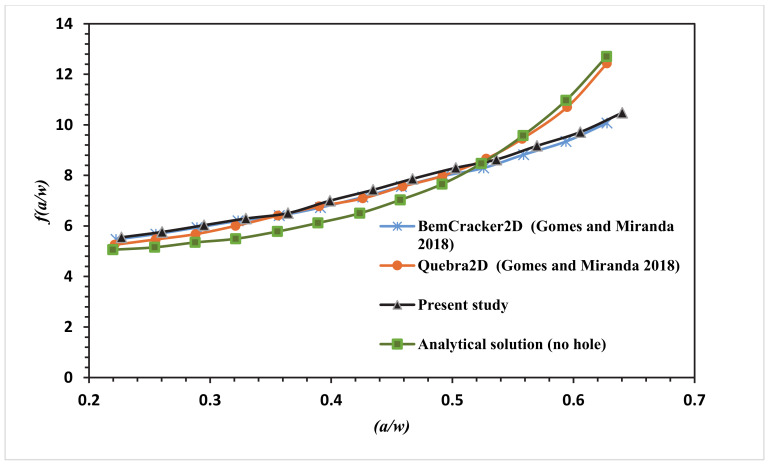
Dimensionless stress intensity factor (SIF) of the modified compact tension specimen CTS01 compared to the work of Gomes and Miranda [16].

**Figure 11:**
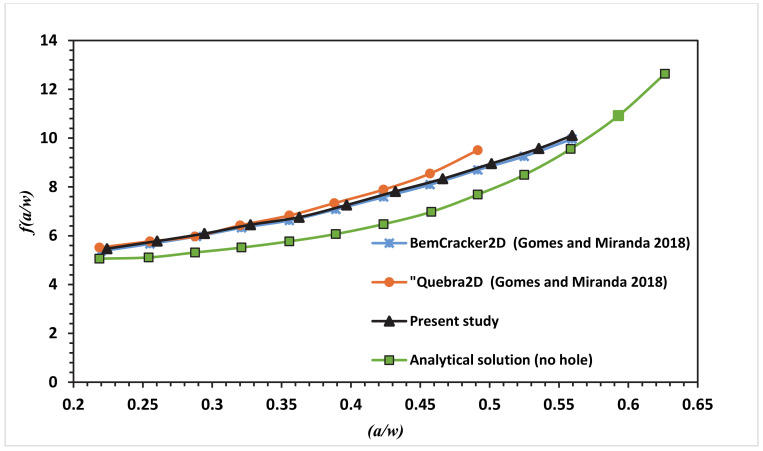
Dimensionless SIF of the modified compact tension specimen CTS02 compared to the work of Gomes and Miranda [16].

**Figure 12:**
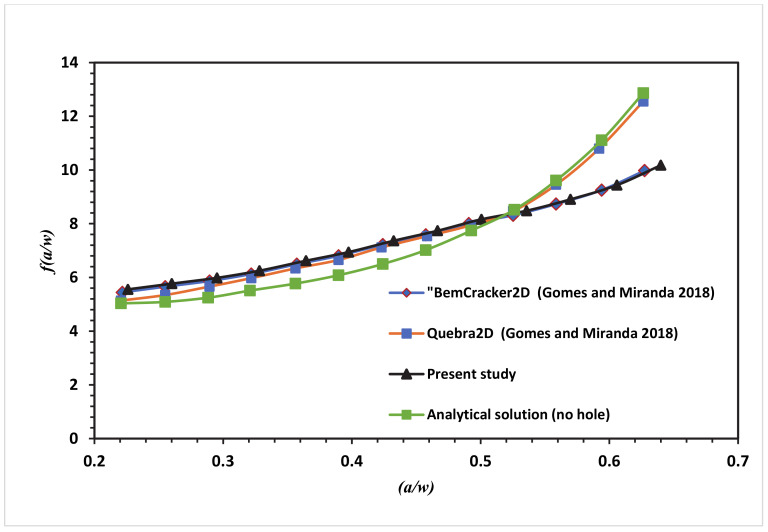
Dimensionless SIF of the modified compact tension specimen CTS03 compared to the work of Gomes and Miranda [16].

**Figure 13:**
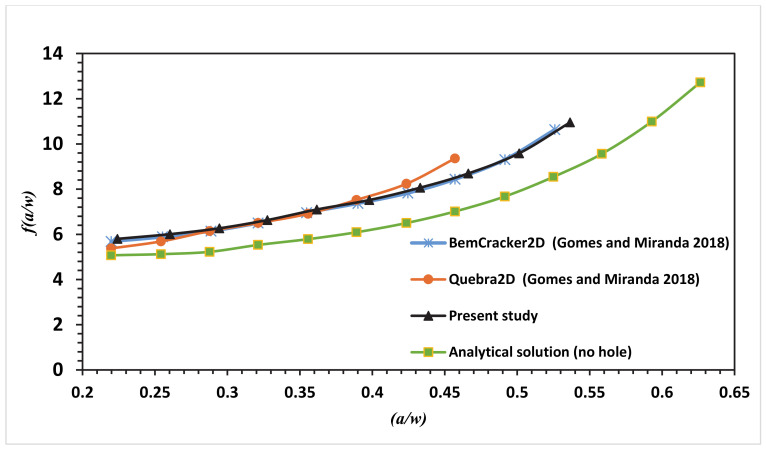
Dimensionless SIF of the modified compact tension specimen CTS04 compared to the work of Gomes and Miranda [16].

The numerical SIF independent of dimension for the MCTS specimen in this analysis is compared with the analytical solution represented in Equation (6) for the CTS without a hole for the four different configurations of the MCTS as shown in Figure 10, Figure 11, Figure 12 and Figure 13. As seen in these figures as the curved crack trajectory established, the *f(a/w)* pattern deviates from each other. Also, the result analysis in present study related to the correction factor *f(a/w)* are analysed with the dimensionless SIF values calculated by (Gomes and Miranda 2018) utilizing the boundary element method (BEM) with BemCracker2D software and the finite element method with Quebra2D (FEM) for the four different configurations as seen in Figure 10, Figure 12 and Figure 13. According to these figures, a strong correlation is observed between the obtained results of the present work and the Quebra2D results when compared to that of BemCracker2D.

In all cases, the crack pathways expected in this work closely match with the results of Gomes and Miranda [16]. Therefore, fatigue crack propagation is also attracted by a hole, so that it can either curve its direction and grow into a hole (sink in the hole’s behaviour) or actually be deflected by a hole and continue to grow when it is ignored (missing the hole’s behaviour). Even if the position of the hole is significantly different, the fatigue life cycles of each structure can vary drastically based on the difference of the crack direction. This also indicates the significance of numerical modelling to simulate these unpredictable fatigue cracks. The simulation results proved that, the fatigue crack was still attracted by the hole dependent of its position from the crack tip, so it could either curve its trajectory and propagate toward the hole or simply deflect at the hole and grow on in its direction.

For the specimens CTS01 and CTS03, the *f(a/w)* curves have approximately the same values up to 0.5 of (a/w), and then the curves start to deviate depending on the different locations of the hole. Furthermore, *f(a/w)* value for CTS04 reached a peak value of 10.7 with a crack length of 20.98 mm immediately prior to the crack sink in the hole, whereas in CTS01, the *f(a/w)* value is 8.8579 with the same crack length of 20.97 mm; thus, the discrepancy between the two *f(a/w)* values for CTS04 and CT01 is 1.8421 with the same crack length of 20.98 mm as shown in Figure 13. The disparity is because of the third hole’s different location. Therefore, in the estimation of a dimensionless stress intensity factor, the third hole location plays a major role.

**Figure 14:**
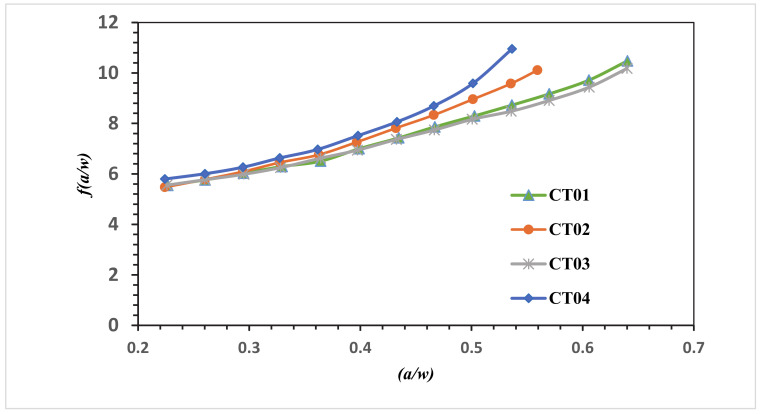
Dimensionless SIF of different configuration of MCTS.

Figure 14 shows the influence of hole position on dimensional stress intensity factor of the different four configurations based on Equations (7)–(10) for CT01, CT02, CT03 and CT 04 respectively.

**Figure 15:**
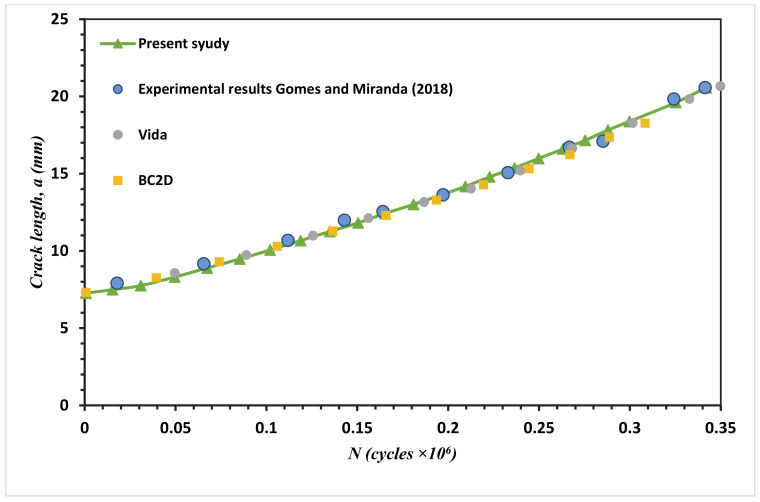
Fatigue life for the MCTS, CTS01.

**Figure 16:**
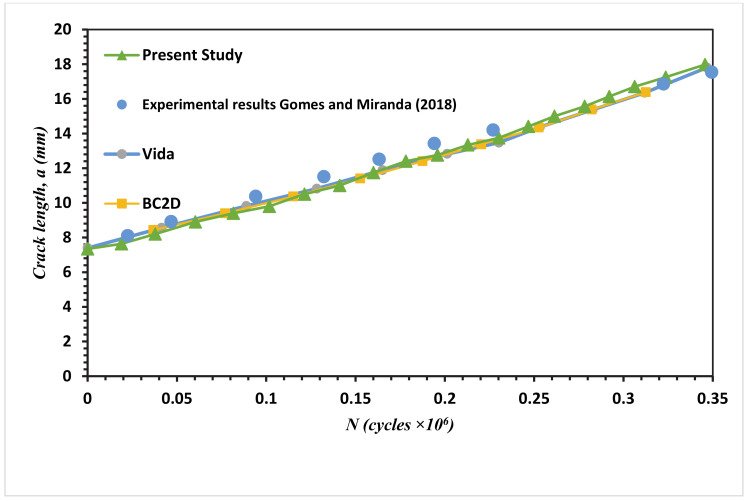
Fatigue life for the MCTS, CTS02.

**Figure 17:**
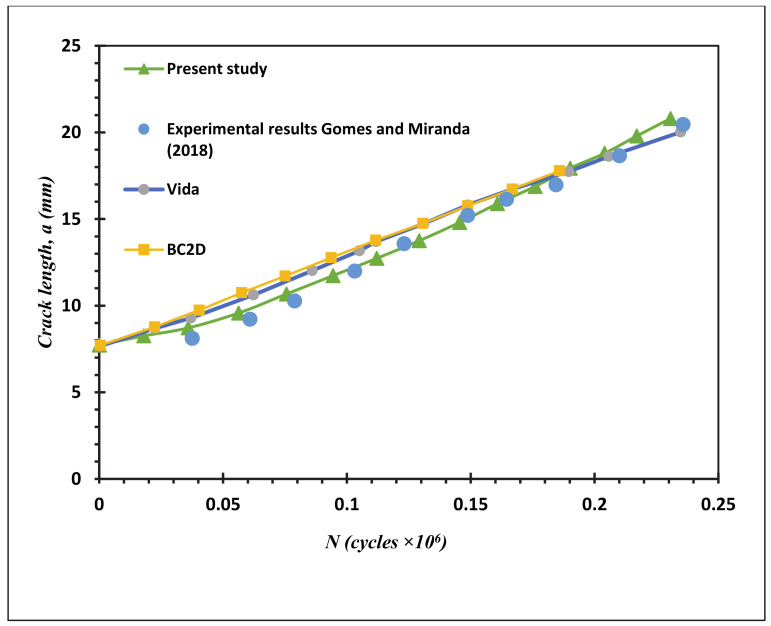
Fatigue life for the MCTS, CTS03.

**Figure 18:**
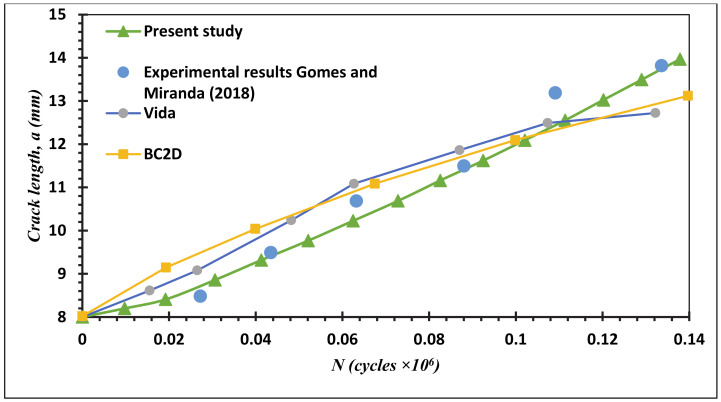
Fatigue life for the MCTS, CTS04.

Fatigue crack growth life estimated using the mixed-mode equivalent SIF is described (Tanaka 1974) as represented in Equation (3). Comparisons between the present study simulated fatigue life results and the experimental results (Gomes and Miranda 2018) for the four different MCTS geometries are shown in Figure 15, Figure 16, Figure 17 and Figure 18 for CT01, CT02, CT03 and CT04, respectively. The simulated FCG life using ANSYS has excellent agreements with the obtained results of Gomes and Miranda [16] as observed in these figures. The present study results were more accurate in the prediction of fatigue life compared to the numerical results obtained by Gomes and Miranda [16] using two software which are VIDA and BemCracker2D (BC2D).

## 4. Conclusions

For MCTS, an extended finite element analysis of mixed-mode fatigue crack propagation was performed using ANSYS Mechanical APDL. The results of XFEM analysis were compared with experimental data for different configurations of MCTS depending on the third hole position from the crack tip with excellent agreement for all cases. The structure and configuration of the specimen play a crucial role in the acquisition of higher values of SIFs in mixed modes which demonstrated the crack growth trajectory. The presence of the hole in the plate influences the crack and changes its path to the hole depending on the location of the hole, so it can either change its way and grow into the hole or it can only be deviated away from the hole and grow. Comparisons with experimental results demonstrate that implementing ANSYS Mechanical APDL 19.2 can predict crack propagation and fatigue life for arbitrary 3D structural components in an effective and economical manner. A best-fit is also proposed for the representation of the dimensionless stress intensity factor for the different configurations of the MCTS based on the numerical data extracted from ANSYS. The best representation of stress distribution was also achieved for all geometries.

## Figures and Tables

**Table 1:** Material properties of SAE 1020 carbon steel.

Property	Value in Metric Unit
Modulus of elasticity, E	205 GPa
Poisson’s ratio, υ	0.29
Yield strength, σ_y_	285 MPa
Ultimate strength, σ_u_	491 MPa
Paris’ law coefficient, C	8.59 × 10^−14^
Paris law exponent m	4.26

**Table 2:** Mesh density based on the number of elements and nodes.

MCTS#	Number of Elements	Number of Nodes
MCTS01	120,097	182,971
MCTS02	130,710	198,991
MCTS03	119,370	181,989
MCTS04	121,409	184,850
